# The PANoptosome: A Deadly Protein Complex Driving Pyroptosis, Apoptosis, and Necroptosis (PANoptosis)

**DOI:** 10.3389/fcimb.2020.00238

**Published:** 2020-06-03

**Authors:** Parimal Samir, R. K. Subbarao Malireddi, Thirumala-Devi Kanneganti

**Affiliations:** Department of Immunology, St. Jude Children's Research Hospital, Memphis, TN, United States

**Keywords:** PANoptosome, PANoptosis, ASC, caspase-1, RIPK1, RIPK3, ZBP1, caspase-8

## Abstract

Programmed cell death is regulated by evolutionarily conserved pathways that play critical roles in development and the immune response. A newly recognized pathway for proinflammatory programmed cell death called PANoptosis is controlled by a recently identified cytoplasmic multimeric protein complex named the PANoptosome. The PANoptosome can engage, in parallel, three key modes of programmed cell death—pyroptosis, apoptosis, and necroptosis. The PANoptosome components have been implicated in a wide array of human diseases including autoinflammatory diseases, neurodegenerative diseases, cancer, microbial infections, and metabolic diseases. Here, we review putative components of the PANoptosome and present a phylogenetic analysis of their molecular domains and interaction motifs that support complex assembly. We also discuss genetic data that suggest PANoptosis is coordinated by scaffolding and catalytic functions of the complex components and propose mechanistic models for PANoptosome assembly. Overall, this review presents potential mechanisms governing PANoptosis based on evolutionary analysis of the PANoptosome components.

## Introduction

**Figure 1:**
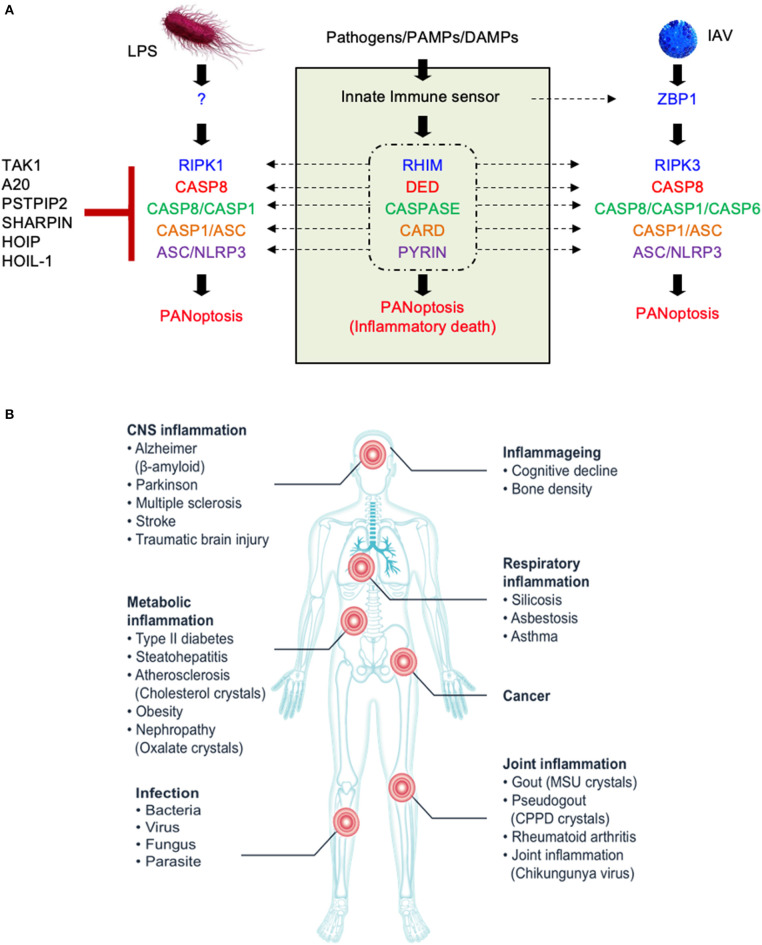
The molecular components of the PANoptosome and the human health significance of PANoptosis. **(A)** Proposed domains of PANoptosome components required for complex assembly in response to various stimuli. PANoptosome assembly is mediated by key molecular motifs collectively called death fold domains. Genetic evidence suggests that PANoptosome activation is inhibited by TAK1, PSTPIP2, SHARPIN, HOIP, HOIL-1, and A20. **(B)** Defects in the PANoptosome components have been implicated in a range of human diseases.

Programmed cell death (PCD) is an evolutionarily conserved process that plays central roles in maintaining organismal homeostasis. Three key PCD pathways have been studied in great detail—pyroptosis (inflammasome-dependent PCD executed by gasdermin family members), apoptosis (PCD mediated by the apoptosome and executioner caspases), and necroptosis (PCD mediated by RIPK3 and the downstream effector MLKL). Previous studies have provided a foundation for us to understand the active and extensive crosstalk between the inflammasome/pyroptosis and apoptosis and necroptosis (Lamkanfi et al., 2008; Malireddi et al., 2010, 2018, 2020; Gurung et al., 2014, 2016a; Lukens et al., 2014; Kuriakose et al., 2016; Zheng et al., 2020). Some pathogenic challenges, such as influenza A virus (IAV) infection (Kuriakose et al., 2016), inhibition of the homeostasis-regulating transforming growth factor β-activated kinase 1 (TAK1) (Malireddi et al., 2018, 2020), and functional alterations in receptor-interacting serine/threonine-protein kinase (RIPK) 1 (Dannappel et al., 2014; Dillon et al., 2014; Kaiser et al., 2014; Rickard et al., 2014a; Dondelinger et al., 2019; Newton et al., 2019a; Lalaoui et al., 2020), can induce pyroptosis, apoptosis, and necroptosis together. These observations raised a question about regulation of PCD in these cases—are the three pathways activated independently of each other, or is a single cell death-inducing complex controlling all three? Studies using mice lacking *Tak1* in myeloid cells and small molecule inhibitors of TAK1 kinase activity suggested the latter (Malireddi et al., 2019, 2020). This evidence led to the identification of a single cell death-inducing complex that is assembled to induce a PCD phenomenon called PANoptosis (Malireddi et al., 2019, 2020). The complex has subsequently been named the PANoptosome (Figure 1A) (Christgen et al., 2020). The PANoptosome was initially shown to contain RIPK1, apoptosis-associated speck-like protein containing a caspase recruitment domain (ASC), nucleotide-binding oligomerization domain (NOD)-like receptor pyrin domain-containing 3 (NLRP3), and caspase (CASP) 8 (Malireddi et al., 2020). A subsequent study determined that RIPK3, CASP6, Z-DNA-binding protein 1 (ZBP1), and CASP1 are also components of the PANoptosome that is assembled in response to IAV infection (Zheng et al., 2020). Collectively, these studies showed that the PANoptosome contains molecules critical for pyroptosis, apoptosis, and necroptosis and is able to activate all three to execute proinflammatory cell death (Christgen et al., 2020). Therefore, PANoptosis provides a mechanism for the host to activate alternative cell death defense mechanisms if one or more of the PCD pathways are compromised by a pathogen or other blockade. In this review, we will discuss PANoptosis and the molecular components of the PANoptosome complex, including their domain structures and functions, and use evolutionary analyses to contextualize the interactions between these molecules.

## Established Mechanisms of PANoptosis

Pathogen sensing by the innate immune system activates intracellular signaling cascades to induce a proinflammatory immune response. Many innate sensors also trigger PCD either by directly interacting with mediators of cell death or through the downstream effects of secreted cytokines. One pathogen that elicits proinflammatory immune responses and PCD is IAV. Yearly seasonal flu outbreaks are caused by IAV strains, but hypervirulent strains sporadically emerge and can result in catastrophic pandemics, such as the 1918 Spanish flu, which killed approximately 50 million people worldwide (Johnson and Mueller, 2002). One feature that made the Spanish flu so deadly was its ability to initiate a dramatic host inflammatory response to the virus, culminating in a rapid “cytokine storm,” severe lung tissue damage, and death. The NLRP3 inflammasome was shown to be a major antiviral host defense mechanism controlling lung inflammation during IAV infection (Kanneganti et al., 2006a,b; Thomas et al., 2009). However, the PCD induced by IAV is not entirely dependent on the NLRP3 inflammasome. The loss of a component essential for just one PCD pathway does not protect the cells from IAV-induced cell death (Kuriakose et al., 2016). Cells lacking the ability to induce pyroptosis, apoptosis, and necroptosis collectively due to genetic loss of *Ripk3* and *Casp8* (or *Fadd*) are protected (Kuriakose et al., 2016). Combinatorial pharmacological inhibition of these three PCD pathways also protects the cells. IAV-induced PCD is also dependent on type I interferon signaling, as cells lacking interferon alpha and beta receptor subunit 1 (IFNAR1) are resistant to IAV-induced cell death (Kuriakose et al., 2016). These findings suggest the existence of upstream regulator(s) controlled by type I interferon signaling. The search for the upstream molecules responsible for NLRP3 inflammasome activation and PCD identified ZBP1—cells lacking *Zbp1* are completely protected from PCD (Kuriakose et al., 2016). Later studies provided additional confirmation that ZBP1 is responsible for RIPK3-dependent cell death during IAV infection (Thapa et al., 2016; Kesavardhana et al., 2017). These observations led to the first description of PANoptosis (Malireddi et al., 2019). Mechanistically, ZBP1 drives PANoptosis by recruiting RIPK3 and CASP8 to the PANoptosome upon sensing IAV infection (Kuriakose et al., 2016; Kesavardhana et al., 2017; Kuriakose and Kanneganti, 2017; Zheng et al., 2020).

Although the ZBP1 PANoptosome promotes PANoptosis to shape the immune response against viral infections, its role in maintaining organismal homeostasis is increasingly being appreciated. Lethality in mice caused by the *Ripk1*^RHIM^ mutation can be rescued by concomitantly deleting ZBP1 or mutating the RHIM domain in ZBP1 (Lin et al., 2016; Newton et al., 2016; Jiao et al., 2020), suggesting that RIPK1 inhibits the aberrant induction of PANoptosis by ZBP1. This evidence extends the concept of PANoptosis beyond the context of infection and shows that it can also regulate fundamental organismal processes such as development.

Further evidence for the role of PANoptosis in organismal processes can be seen from studies with RIPK1. The regulation of PANoptosis by RIPK1 is essential for homeostasis, cell death, and inflammatory immune responses. The scaffolding function of RIPK1 promotes cell survival signaling via the assembly of complex-I, which blocks both early and late cell death checkpoints downstream of TNFR1 signaling (Gerlach et al., 2011; Vince et al., 2012; Dondelinger et al., 2013; Peltzer et al., 2016, 2018; Ting and Bertrand, 2016; Taraborrelli et al., 2018), while RIPK1's kinase activity or receptor-interacting protein homotypic interaction motif (RHIM) function enables PCD (Dillon et al., 2014; Kaiser et al., 2014; Rickard et al., 2014b; Dowling et al., 2015; Newton et al., 2019a; Lalaoui et al., 2020; Tao et al., 2020). Deletion of RIPK1 in mice is embryonically lethal, which may be caused by the activation of RIPK3-regulated PANoptosis-like cell death through CASP8 and Fas-associated protein with a death domain (FADD) leading to systemic inflammation (Kelliher et al., 1998; Zhang et al., 2011; Dillon et al., 2014). In addition, mutations inactivating CASP8 catalytic activity lead to embryonic lethality in mice mediated by PANoptosis through the activation of RIPK1, RIPK3-MLKL, and CASP1 (Fritsch et al., 2019; Newton et al., 2019b). Mutating the CASP8 cleavage site in RIPK1, which is essential to regulate RIPK1 activity, also causes embryonic lethality and inflammation (Newton et al., 2019a; Lalaoui et al., 2020; Tao et al., 2020). Collectively, these studies provide evidence for the regulation of PANoptosis by RIPK1 and CASP8.

Another key regulator of PANoptosis was identified through a study of the complex-I component TAK1 (Malireddi et al., 2018). TAK1 was found to play an important role in NLRP3 inflammasome activation and to also modulate RIPK1 activity to regulate PANoptosis (Malireddi et al., 2019). In the absence of TAK1 activity due to pharmacological inhibition or genetic loss, RIPK1 activates PANoptosis and inflammation through its kinase activity (Malireddi et al., 2018; Orning et al., 2018; Sarhan et al., 2018). A follow up study discovered RIPK1 kinase-independent activation of PANoptosis, questioning the dogma that RIPK1 kinase function is required for PCD (Malireddi et al., 2020). These studies suggest that loss of function or deregulation of RIPK1 homeostasis can engage PANoptosis and affect organismal development and immune responses.

The discovery of PANoptosis in the contexts of IAV infection and organismal homeostasis led us to revisit autoinflammatory diseases in which components of the PCD machinery have been implicated to determine whether PANoptosis also plays a role here. A mutation in the *Pstpip2* gene (*Pstpip2*^cmo^) causes autoinflammation via CASP8- and NLRP3/CASP1-regulated PCD. Combined deletion of *Ripk3, Casp1*, and *Casp8*, which blocks all three arms of PANoptosis (pyroptosis, apoptosis, and necroptosis), rescued the mice from the autoinflammatory condition, while inhibition of a single arm was not sufficient for rescue (Lukens et al., 2014; Gurung et al., 2016a), suggesting the involvement of PANoptosis in mediating the autoinflammatory condition. Another example of PANoptosis in autoinflammation can be seen in mice with a mutation in *Sharpin* (*Sharpin*^cpdm^). The inflammation in *Sharpin*^cpdm^ mice can be rescued by deletion of TNF or expression of kinase-dead RIPK1 (Gerlach et al., 2011; Berger et al., 2014). It can also be rescued by hemizygous deletion of CASP8 along with the loss of RIPK3 (Rickard et al., 2014b). Skin inflammation can also be delayed by the loss of *Nlrp3* or the inflammasome-dependent cytokine IL-1β (Berger et al., 2014; Douglas et al., 2015; Gurung et al., 2016b). Involvement of the three arms of PANoptosis suggests a role for PCD and the inflammatory cytokines released by PANoptotic cells in driving inflammation in *Sharpin*^cpdm^ mice.

Several other proteins associated with autoinflammation with dual roles in cell survival and inflammatory immune signaling pathways have been implicated in PANoptosis. In addition to RIPK1, deletion of other complex-I molecules, including HOIP, HOIL-1, and A20, also leads to embryonic lethality and autoinflammatory diseases, suggesting they may have a role in regulating PANoptosis (Gerlach et al., 2011; Vince et al., 2012; Dondelinger et al., 2013; Vande Walle et al., 2014; Peltzer et al., 2016; Ting and Bertrand, 2016; Taraborrelli et al., 2018; Zhang et al., 2019).

Whether it is induced in response to infection, during organismal homeostasis, or in the context of autoinflammation, PANoptosis is executed by a molecular complex called the PANoptosome that integrates apoptotic, necroptotic, and inflammasome components. Downstream cytokines such as IL-1β and IL-18 and damage-associated molecular patterns (DAMPs) released by PANoptotic cells act as alarmins initiating and amplifying the inflammatory response. Overall, the PANoptosome is responsible for driving this inflammatory form of cell death, and defects in PANoptosome components have been implicated in a host of human diseases including autoinflammatory and neurodegenerative diseases, cancer, and susceptibility to pathogenic infections (Figure 1B, Table 1).

**Table 1:** A list of PANoptosome components and associated human diseases.

**PANoptosome component**	**Associated disease(s)**	**References**
*NLRP3*	Cryopyrin-associated periodic syndrome, Familial cold urticaria, Muckle-Wells syndrome	Hoffman et al., 2001; Aganna et al., 2002; Dodé et al., 2002; Rösen-Wolff et al., 2003; Hawkins et al., 2004; Verma et al., 2010; Kuemmerle-Deschner et al., 2011; Rowczenio et al., 2013; Alejandre et al., 2014
	Chronic infantile neurological cutaneous and articular syndrome	Aksentijevich et al., 2002; Feldmann et al., 2002; Neven et al., 2004; Jesus et al., 2008
	Crohn's disease	Schoultz et al., 2009; Zhang H. et al., 2014
*RIPK3*	Steatohepatitis	Roychowdhury et al., 2013; Wang et al., 2016
*RIPK1*	Adenocarcinoma of large intestine	Greenman et al., 2007
*ASC*	Liver carcinoma	Zhang et al., 2007
	Colorectal carcinoma	Yokoyama et al., 2003; Ohtsuka et al., 2006; Riojas et al., 2007
	Non-small cell lung carcinoma	Rosell et al., 2002; Virmani et al., 2003; Machida et al., 2006; Zhang et al., 2006
*CASP1*	Multiple sclerosis	Furlan et al., 1999; Huang et al., 2004; Peelen et al., 2015
*CASP8*	Autoimmune lymphoproliferative syndrome type 2B	Chun et al., 2002
	Breast carcinoma	MacPherson et al., 2004; Michailidou et al., 2017
	Liver carcinoma	Soung et al., 2005
*TAK1*	Cardiospondylocarpofacial syndrome	Le Goff et al., 2016
	Frontometaphyseal dysplasia	Wade et al., 2016
	Malignant neoplasm of prostate	Liu et al., 2007
*A20*	Autoinflammatory syndrome, familial, Behcet-like	Shigemura et al., 2016; Zhou et al., 2016
	Rheumatoid arthritis	Lodolce et al., 2010; Stahl et al., 2010; Matmati et al., 2011; Lee et al., 2012; Perkins et al., 2012; Prahalad et al., 2013; Hao et al., 2014; Kim et al., 2014; Zhang X. et al., 2014; Hoffjan et al., 2015; Shen et al., 2017
	Systemic lupus erythematosus	Adrianto et al., 2011; Fan et al., 2011
	Psoriasis	Nair et al., 2009; Strange et al., 2010; Jiang et al., 2012; Tsoi et al., 2012; Indhumathi et al., 2015
	Sjögren's syndrome	Sisto et al., 2011
*HOIL-1*	Polyglucosan body myopathy 1 with or without immunodeficiency	Nilsson et al., 2013
	Rhabdomyolysis	Wang et al., 2013
*PSTPIP2*	Chronic recurrent multifocal osteomyelitis	El-Shanti and Ferguson, 2007; Ferguson and El-Shanti, 2007

## Components of the PANoptosome

Genetic and biochemical data suggest that proteins that are part of the PANoptosome can be generally categorized into three classes—(1) ZBP1 and NLRP3 as putative PAMP and DAMP sensors, (2) ASC and FADD as adaptors, and (3) RIPK1, RIPK3, CASP1, and CASP8 as catalytic effectors. A recent study identified CASP6 as a critical component of the PANoptosome that promotes its assembly by reinforcing the interaction between RIPK3 and ZBP1 in the complex, suggesting a novel scaffold function for this presumed apoptotic executioner caspase (Zheng et al., 2020). Whether the catalytic activity of CASP6 is required for PANoptosome assembly remains to be fully understood. Additionally, classification of RIPK1 presents a challenge. Earlier studies had shown that RIPK1 kinase activity was required for the induction of necroptosis. However, a more recent study showed that RIPK1 is recruited to the PANoptosome, and the kinase-dead mutant of RIPK1 is capable of inducing PANoptosis, suggesting that RIPK1 may act as an adaptor in some cases and a catalytic effector in others (Malireddi et al., 2020). These observations suggest that PANoptosome components might have multiple biochemical functions involved in the execution of PANoptosis.

## The Inflammasome, Apoptosome, and Necroptosome as Templates for Panoptosome Formation

PANoptosome assembly likely requires domain-specific homotypic and heterotypic interactions between the molecular components. A closer look at the molecular mechanisms of PCD makes it clear that multimeric protein interactions and catalytic activities constitute two major functionally important aspects to induce cell death. In the case of pyroptosis, cell death is executed by a hetero-multimeric complex called the inflammasome (Martinon et al., 2002; Kanneganti et al., 2006a; Mariathasan et al., 2006; Sutterwala et al., 2007; Bürckstümmer et al., 2009; Fernandes-Alnemri et al., 2009; Hornung et al., 2009; Broz and Dixit, 2016). Inflammasome assembly involves homotypic interactions among the caspase recruitment domains (CARDs) and PYRIN domains (PYDs) of inflammasome sensors, the adaptor ASC, and the catalytic actuator CASP1 (Man and Kanneganti, 2015; Broz and Dixit, 2016). Intrinsic apoptosis is driven by another hetero-multimeric complex known as the apoptosome. Apoptosome assembly exploits the homotypic interactions of CARDs for assembly and activation of the catalytic actuator CASP9 for cell death execution (Zou et al., 1999; Acehan et al., 2002; Bao and Shi, 2007; Riedl and Salvesen, 2007; Zmasek and Godzik, 2013). Extrinsic apoptosis induced by death receptors exploits the homotypic interactions mediated by death effector domains (DEDs) and death domains (DEATH) to assemble the death-inducing signaling complex (DISC), which activates the cysteine protease CASP8 (Lo et al., 2015; Yang, 2015). In necroptosis, the molecular mechanism of assembly and the molecular composition of the necroptosome are not entirely clear, but available data suggest that assembly might require the homotypic interactions of RHIMs and the kinase activity of RIPK3 for cell death execution (Wallach et al., 2011; Li et al., 2012; Vanden Berghe et al., 2016; Zhang et al., 2016; Mompeán et al., 2018; Weber et al., 2018). The PANoptosome likely combines many of the same interactions as the inflammasome, DISC, and necroptosome and acts as a versatile PCD complex utilizing necroptosome-associated kinase, as well as apoptosis- and pyroptosis-associated protease, activities for cell death execution.

## Evolution of the PANoptosome Components

Genetic studies have established that PANoptosome components have evolved to play key roles in cellular homeostasis, development, and inflammatory immune responses (Lin et al., 2016; Newton et al., 2016, 2019a,b; Malireddi et al., 2018; Fritsch et al., 2019; Kesavardhana et al., 2020; Lalaoui et al., 2020). Although some of the conserved molecular motifs present in cell death-executing complexes exist in all branches and kingdoms of life, others are present only in higher eukaryotes and viruses. The key molecules involved in pyroptosis, apoptosis, and necroptosis share these domains, which indicates that the three pathways employ similar assembly mechanisms for large hetero-multimeric cell death-inducing complexes.

**Figure 2:**
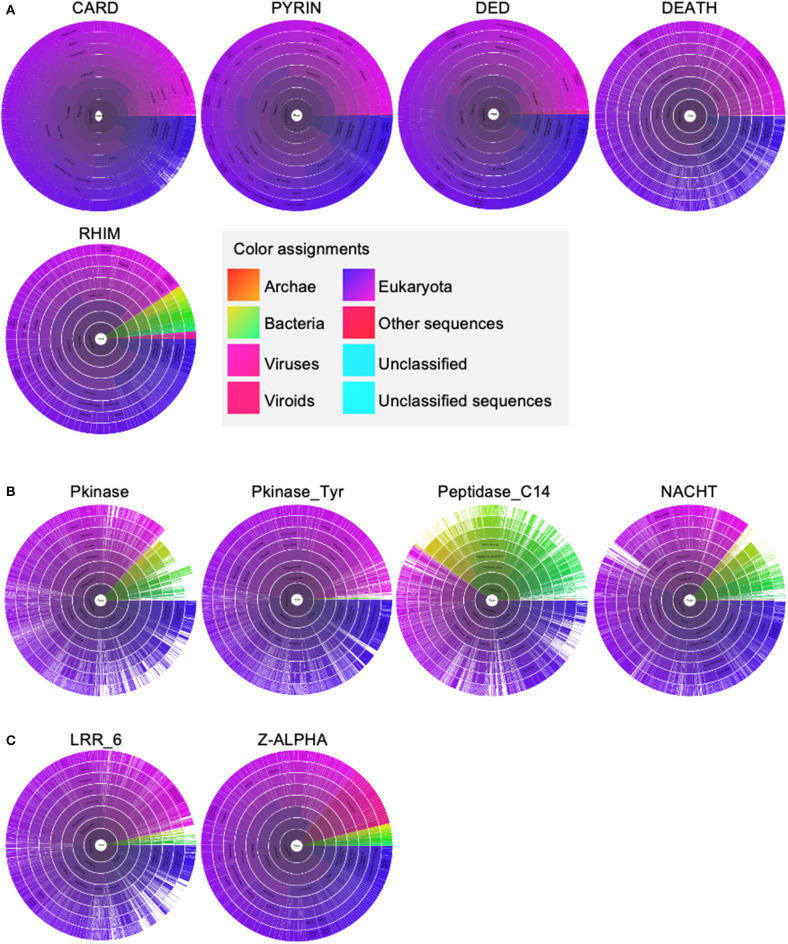
The distribution of molecular motifs present in PANoptosome components. **(A)** Distribution of assembly domains. **(B)** Distribution of catalytic domains. **(C)** Distribution of sensing domains. All domain names and their distributions were obtained from the Pfam database (Sonnhammer et al., 1997). Sunburst visualizations and color assignment legends were exported from the Pfam database.

To develop a framework to understand the interactions between these molecules, we performed an evolutionary analysis to help identify important domains for PANoptosome assembly because the critical domains are more likely to coevolve. We separated the domains of the PANoptosome components into three classes—(1) assembly domains, (2) catalytic domains, and (3) sensing domains. Assembly domains include CARD, DEATH, DED, PYD, and RHIM. Catalytic domains are Peptidase_C14 (caspase domain), Pkinase (kinase domain in RIPK3), Pkinase_Tyr (kinase domain in RIPK1), and NACHT (ATPase domain in NLRP3). Sensing domains include z-alpha and LRR_6. We used the Pfam database to look at the evolution of these domains (Sonnhammer et al., 1997). Four of the assembly domains (CARD, DEATH, DED, and PYD) are also called death fold domains due to their critical role in assembling PCD-executing complexes. The death fold domains seem to be present only in multicellular organisms and a few viruses that infect them (Figure 2A). Although Pkinase_Tyr, RHIM, z-alpha, and LRR_6 domains do exist in prokaryotes, most of the proteins containing these domains are present in eukaryotes with a few exceptions in viruses (Figures 2A–C). Pkinase, Peptidase_C14, and NACHT domains are more widely distributed among the kingdoms of life (Figure 2B). These data suggest that some of the molecular motifs responsible for catalytic functions might have been present in the common ancestors of eukaryotes and prokaryotes. However, a functional PANoptosome might have coevolved with the development of multicellularity because the death fold domains that seem to be essential for its assembly are present mainly in metazoans. This is in line with the role of PANoptosis in modulating organismal homeostasis (Dillon et al., 2014; Malireddi et al., 2018, 2019; Newton et al., 2019a; Lalaoui et al., 2020).

## The Mechanism of PANoptosome Assembly Through Phylogenetic Analysis

**Figure 3:**
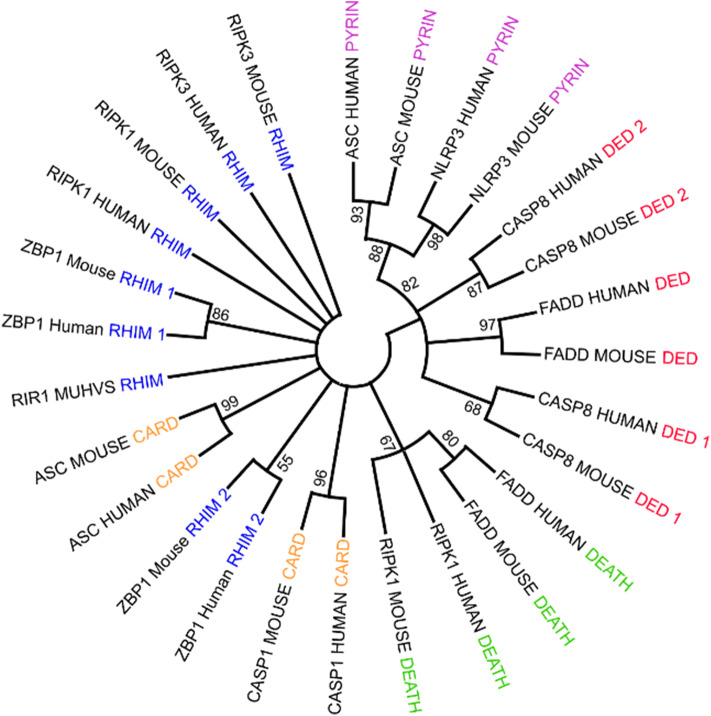
Using phylogenetic analysis to understand the molecular mechanism governing PANoptosome assembly. Phylogenetic analysis of assembly domains present in PANoptosome components. Amino acid sequences corresponding to the domains were obtained from the Uniprot database (The UniProt Consortium, 2015). Multiple sequence alignment and phylogenetic analysis were performed using the Mega-X software package (Kumar et al., 2018). ClustalW was used for multiple sequence alignment. Maximum likelihood analysis with bootstrap 500 iterations was used to generate the phylogenetic tree. The consensus phylogenetic tree is shown. If a protein contains multiple copies of a domain, the domains are numbered beginning at the N-terminus. For example, the first occurrence of the RHIM domain in ZBP1 is named RHIM 1, and the second occurrence is named RHIM 2.

PANoptosome components contain a wide range of molecular motifs that can be utilized for assembly of the active complex. However, a clear domain-domain homotypic interaction-based assembly mechanism has not yet been deciphered. Phylogenetic analysis of the assembly domains of the components using Maximum Likelihood Analysis allows a testable model for the mechanism of PANoptosome assembly to be built (Figure 3). Since the homotypic interactions between the assembly domains are associated with sequence similarity between them, the domains capable of heterotypic interactions would hypothetically be closer together in a phylogenetic tree. Phylogenetic analysis revealed that one of the DED motifs in CASP8 (DED2^CASP8^) is in the same branch as the PYD from NLRP3 and ASC. Previous studies have revealed that the PYD of ASC (PYD^ASC^) is required for CASP8 polymerization potentially through a heterotypic interaction between DED2^CASP8^ and PYD^ASC^ (Vajjhala et al., 2015), corroborating the phylogenetic data. The CASP8 DED1 motif (DED1^CASP8^) was close to the FADD DED motif (DED^FADD^). The homotypic interaction mediated by DED^FADD^ and DED1^CASP8^ is known to be important for DISC assembly during extrinsic apoptosis (Kischkel et al., 1995; Muzio et al., 1996; Fu et al., 2016). Another feature of the phylogenetic tree was that the CARD domain of ASC (CARD^ASC^) and one of the RHIM domains of ZBP1 (RHIM2^ZBP1^) were closer to each other than to CARD^ASC^ and CARD^CASP1^ or RHIM1^ZBP1^ and RHIM2^ZBP1^, respectively. This result suggests that upon ligand sensing, ZBP1 may be able to induce assembly of the PANoptosome by recruiting ASC through a heterotypic interaction between RHIM2^ZBP1^ and CARD^ASC^.

These observations suggest a putative assembly mechanism for the ZBP1 PANoptosome involving IAV vRNP sensing by ZBP1 (Figure 3). In this model, activated ZBP1 would bind to ASC through RHIM2^ZBP1^ and CARD^ASC^ heterotypic interactions and induce ASC polymerization. PYD^ASC^ can then be engaged to recruit NLRP3 into the complex through PYD^NLRP3^. Since CASP1 activation in IAV-infected cells is strictly dependent on NLRP3, NLRP3 recruitment to the PANoptosome must precede recruitment of CASP1 (Kanneganti et al., 2006b). In cells lacking TAK1 kinase activity, CASP1 activation is again dependent on NLRP3, but the identity of the upstream sensor that induces assembly of the PANoptosome in these cells remains unclear (Malireddi et al., 2018). This sequence of events suggests that there may be a conformational change in CARD^ASC^ after PYD^NLRP3^ binding to PYD^ASC^ that would allow CASP1 binding. The change in binding capacity could also be due to an increase in valency because the NACHT domain of NLRP3 (NACHT^NLRP3^) increases PYD^ASC^ polymerization (Cai et al., 2014; Lu et al., 2014; Samir and Kanneganti, 2019). While the RHIM2^ZBP1^ valency is occupied to build the ASC-mediated portion of the complex, the free RHIM valency (RHIM1^ZBP1^) can recruit RIPK1 and/or RIPK3. CASP8 can be recruited to the complex in two ways—(1) through the DED domain of RIPK1 (DED^RIPK1^) interacting with one of the DED^CASP8^ domains and/or (2) through PYD^ASC^ interacting with DED2^CASP8^. Future experiments will help define the specific molecular interactions facilitating PANoptosome assembly.

## Alternative Assembly Strategies

Two recent studies have suggested a role for interactions mediated by intrinsically disordered regions (IDR) in inducing PCD (Samir et al., 2019; Zheng et al., 2020). In one study, the interaction between NLRP3 and DDX3X was proposed to be IDR-mediated and was critical for pyroptosis downstream of NLRP3 inflammasome activation (Samir et al., 2019). In another study, CASP6 was found to promote IAV-induced PANoptosis dependent on an IDR-mediated interaction between CASP6 and RIPK3. This interaction increased the efficiency of RIPK3 recruitment to the ZBP1-induced PANoptosome (Zheng et al., 2020). The possibility of IDR involvement in the assembly of the PANoptosome increases the range of available interaction modalities because all known PANoptosome components have predicted IDRs. Additionally, some of the assembly domains have the ability to undergo prionoid phase transitions (Cai et al., 2014; Lu et al., 2014; Li et al., 2018). Therefore, it is likely that assembly of the PANoptosome can lead to signal amplification through polymerization and prionoid phase transitions, resulting in the assembly of large cytoplasmic supramolecular structures that can be visualized using immunofluorescence microscopy.

The PANoptosome contains molecules that can induce PCD by multiple mechanisms. This means that a full complex may not need to be assembled before cells undergo cell death. This provides opportunities, and difficulties, for the molecular dissection of PANoptosis. For example, inhibition of one arm of PANoptosis will not be sufficient to prevent cells from dying. This can be exploited to study the ways in which different PCD pathways crosstalk with each other. Additionally, identification of apical sensors that control PANoptosis as a whole will be critical for the modulation of this process. So far ZBP1 is the only apical sensor that has been identified to induce PANoptosis in response to IAV infection (Kuriakose et al., 2016; Kesavardhana et al., 2017). However, TAK1 inhibition-induced PANoptosis provides compelling evidence for the existence of alternative modes of PANoptosis activation that do not depend on the ZBP1 PANoptosome but instead result from pathogen-induced cellular perturbations being detected as danger signals, triggering the activation of this highly inflammatory form of cell death (Malireddi et al., 2018, 2020; Orning et al., 2018; Sarhan et al., 2018). These studies indicate that PANoptosis has evolved as a robust fail-safe mechanism to induce inflammatory cell death and promote protective antimicrobial immune responses, especially in response to pathogens that target key inflammatory signaling cascades or cell death pathways (Philip et al., 2014; Weng et al., 2014; Malireddi et al., 2018, 2019, 2020; Orning et al., 2018; Sarhan et al., 2018). It remains to be seen if there are additional sensors that are activated in response to other pathogenic challenges to induce PANoptosis. It is possible that other apical sensors for PANoptosis do not engage exactly the same molecular machinery as ZBP1 or TAK1 inhibition, allowing PANoptosomes with differing compositions to form. This would be in line with the lessons learned from pyroptosis and inflammasomes, as the sensors NLRP1b, NLRP3, and NLRC4 have differential requirements for the adaptor ASC when assembling the inflammasome (Case et al., 2009; Abdelaziz et al., 2011; De Jong et al., 2014; Opdenbosch et al., 2014; Patankar et al., 2015; Broz and Dixit, 2016; Li et al., 2018). The ZBP1 PANoptosome can be thought of as the founding member of the PANoptosome class of PCD-inducing complexes, and evidence suggests there are additional forms of the PANoptosome (Malireddi et al., 2018, 2020; Christgen et al., 2020).

## Summary

The recent progress in our understanding of the extensive crosstalk between different cell death and signaling cascades unequivocally establishes the existence of multifaceted signaling platforms. The PANoptosome represents one such platform which can engage multiple cell death modalities including pyroptosis, apoptosis, and necroptosis to form the collective inflammatory cell death pathway of PANoptosis. The PANoptosome appears to have evolved to efficiently utilize diverse modes of cellular communication that include domain-based scaffolds and enzyme-based catalytic modifications to engage PANoptosis. Using these diverse cellular mechanisms might be an important way in which the PANoptosome incorporates fundamentally different modes of cell death to orchestrate homeostasis during developmental programs while driving a robust inflammatory form of cell death for immune-mediated protection against microbial infections. Because many of the PANoptosome components have been associated with disease, including autoinflammatory, infectious, and neurodegenerative diseases and cancer, improved understanding of the molecular underpinnings of the PANoptosome will be able to inform the development of targeted inhibitors and activators of inflammatory cell death for therapeutic modulation of inflammation and the immune response.

## Author Contributions

PS performed the phylogenetic and evolutionary analyses. All authors contributed to writing and editing the manuscript.

## Conflict of Interest

The authors declare that the research was conducted in the absence of any commercial or financial relationships that could be construed as a potential conflict of interest.
